# Sarcoidosis data-driven patient trajectories and predictors of chronic disease

**DOI:** 10.1186/s12931-026-03830-z

**Published:** 2026-07-24

**Authors:** Marios Rossides, Susanna Kullberg, Pernilla Darlington, Anders Eklund, Elizabeth V. Arkema

**Affiliations:** 1 Unit of Epidemiology, Institute of Environmental Medicine, Karolinska Institutet, Stockholm, Sweden; 2 Department of Respiratory Medicine and Allergy, Theme Inflammation and Ageing, Karolinska University Hospital, Gävlegatan 55, Stockholm, 171 76 Sweden; 3 Division of Immunology and Respiratory Medicine, Department of Medicine Solna; Center for Molecular Medicine, Department of Medicine Solna, Karolinska Institutet, Stockholm, Sweden; 4 Department of Internal Medicine, Södersjukhuset, Stockholm, Sweden; 5 Department of Clinical Science and Education, Karolinska Institutet, Södersjukhuset, Stockholm, Sweden; 6 Clinical Epidemiology Division, Department of Medicine Solna, Karolinska Institutet, Stockholm, Sweden

**Keywords:** Sarcoidosis, Chronic disease, Risk factors, Complications, Registries, Sweden

## Abstract

**Background:**

Sarcoidosis is a heterogeneous granulomatous disease with an unpredictable course. Population-level evidence describing its long-term trajectories and their prognostic implications remains limited.

**Methods:**

We identified individuals newly diagnosed with sarcoidosis in the Swedish National Patient Register (≥ 2 ICD-coded visits; 2006–2018). Sarcoidosis-related visits over five years were modeled using zero-inflated Poisson finite mixture models to identify trajectories. Baseline demographic and clinical predictors of trajectory membership were assessed with Poisson regression. Associations between trajectories and long-term outcomes were estimated using multivariable Cox regression. Supplementary analyses incorporated clinical, genetic, and physiological data from the Karolinska cohort.

**Results:**

Among 9665 patients, two distinct trajectories were identified: a resolving trajectory (71.5%) with near-complete remission of sarcoidosis-related visits within two years, and a chronic trajectory (28.5%) with persistently elevated visit rates over five years. Older age modestly increased the risk of chronic disease. The strongest predictor of chronic disease was immunosuppressive treatment around diagnosis (risk ratio 2.18 [95% CI 2.04, 2.33]). Diagnosis in neurology, ophthalmology, or cardiology, uveitis, and hospitalization at diagnosis were also associated with chronicity, whereas diagnosis in rheumatology and dispensation of non-steroidal anti-inflammatory drugs were protective. In the Karolinska cohort, Löfgren’s syndrome and HLA-DRB1*03 were strongly associated to a resolving course. Chronic sarcoidosis was associated with higher risks of infection, heart failure, diabetes, depression, anxiety, and early death.

**Conclusions:**

Sarcoidosis follows two long-term trajectories. Clinical phenotype and need of early treatment predict chronicity, which is associated with substantial long-term morbidity and mortality. Early prognostication may support personalized sarcoidosis management.

**Supplementary Information:**

The online version contains supplementary material available at 10.1186/s12931-026-03830-z.

## Background

Sarcoidosis is a systemic granulomatous disease of unknown etiology that involves the lungs in 90% of cases [1]. The clinical course of sarcoidosis varies greatly and is largely unpredictable at diagnosis [2]. Findings of mostly clinical studies that focused on pulmonary disease suggest that inflammation and signs of disease subside or resolve in about 60–70% of patients within two to five years after diagnosis irrespective of treatment, whereas sarcoidosis may persist in the rest (chronic sarcoidosis) [3–7]. Progressive disease may lead to organ function decline and early death in some patients [1]. It remains unknown whether this dimorphic clinical course of sarcoidosis can be replicated in large population-based cohorts and to what extent the risk of unfavorable outcomes affects individuals with chronic sarcoidosis [7].

No single sarcoidosis-related factor or biomarker can accurately prognosticate the course of disease at diagnosis [8]. It is widely accepted that Löfgren’s syndrome, a phenotype of sarcoidosis diagnosed in about 30% of patients in Scandinavia is self-resolving in 80–90% of cases, particularly patients who carry human leukocyte antigen (HLA)-DRB1*03 alleles [9–11]. Additionally, the need for immunosuppressant treatment, which is recommended in patients with debilitating symptoms or organ function deterioration, has been used in studies to characterize patients with progressive or chronic disease who might be at higher risk of complications and early death [6, 7, 12, 13]. The utility of this definition in predicting a chronic course for sarcoidosis has not been sufficiently tested.

The main purpose of this study was to identify the five-year trajectories of patients with sarcoidosis in a large register-based cohort using a data-driven approach. In addition, we aimed to examine whether demographic and/or sarcoidosis-related factors evaluated at diagnosis were predictive of the data-derived disease trajectories and if these trajectories were associated with long-term adverse outcomes after diagnosis.

## Methods

### Study setting and data sources

We conducted a cohort study using information from Swedish health and administrative registers. In the Swedish tax-funded and universally accessible healthcare system, interactions with healthcare services (e.g., hospitalizations, outpatient visits, dispensations of prescribed medications) are collected in registers. The National Patient Register (NPR) records healthcare visits on a nationwide basis (inpatient since 1987, outpatient since 2001); diagnoses are coded using revisions of the International Classification of Diseases (ICD). All dispensations of prescribed medications in pharmacies across the country, marked by their Anatomical Therapeutic Chemical (ATC) code, are available in the National Prescribed Drug Register (NPDR) since July 2005. Purchase of over-the-counter medications and hospital-administered treatments are not captured. Register records can be linked using an individual’s unique identification number, which is allocated at birth or immigration.

### Study population

We used a previously validated definition to identify individuals with sarcoidosis in the NPR (positive predictive value 94%) [14]. We required ≥ 2 inpatient or outpatient visits listing an ICD code for sarcoidosis as either the primary or one of the secondary discharge diagnoses from January 1, 2006 to December 31, 2018 (codes in Table S1). Limiting inclusion to 2006–2018 was necessary to examine dispensations of treatments in the NPDR and to ensure all patients could be followed for up to five years. The date of first visit was considered the date of diagnosis.

To minimize misclassification, we excluded: (a) individuals with a hematopoietic or lung malignancy recorded in the National Cancer Register within six months before or after diagnosis of sarcoidosis (codes in Table S1), (b) those who immigrated to Sweden less than two years before sarcoidosis diagnosis (dates from the Total Population Register), and (c) individuals < 18 or > 85 years old at sarcoidosis diagnosis.

### Covariates and predictors

We used register data to define sociodemographic and clinical variables that may be predictive of sarcoidosis trajectories. Sociodemographic variables were evaluated at sarcoidosis diagnosis. From the Total Population Register, we obtained birth date (to calculate age), sex (female/male), region of residence (grouped into healthcare regions Stockholm [including Gotland], Uppsala-Örebro, West, South, Southeast, or North) [15], Nordic country of birth, and civil status (married or in registered partnership, or other [e.g., unmarried, divorced, or widowed]). From the Longitudinal Integrated Database for Health Insurance and Labor Market Studies, we assessed attained education (≤9 years, 10–12, ≥13 years, or missing).

Using data from the NPR, we assessed whether patients were hospitalized at sarcoidosis diagnosis and used the clinic’s specialization to approximate the primary manifestation of sarcoidosis at diagnosis. Clinics were grouped into respiratory medicine, rheumatology, cardiology, other medicine (primarily internal medicine which may include respiratory clinics in some smaller hospitals), dermatology, neurology, ophthalmology and other (e.g., surgery, acute medicine). We counted visits in the NPR within two years before sarcoidosis diagnosis as a measure of patients’ general health. Family history of sarcoidosis was defined as having ≥ 1 first-degree relative (identified in the Multi-Generation Register) with sarcoidosis at the time of diagnosis (Table S1). We assessed comorbidities within up to three months before diagnosis (to reduce surveillance bias [15]) using data from the NPR and NPDR as outlined in Table S1: myocardial infarction, heart failure, atrial fibrillation, hypertension, diabetes mellitus, chronic obstructive pulmonary disease, asthma, uveitis, autoimmune disease, primary (non-acquired) immunodeficiency, and infectious disease; the latter within six months before diagnosis.

We classified patients who were dispensed ≥1immunosuppressant treatment in the NPDR (one of systemic corticosteroid [first line], methotrexate, or azathioprine [common second-line treatments in Sweden]) ±3 months from sarcoidosis diagnosis as treated for sarcoidosis around the time of diagnosis. Immunosuppressive treatment is recommended in patients with debilitating symptoms or poor quality of life, manifestations affecting vital organs or organ function, or presumed progressive disease [16]. We have used this as a marker of sarcoidosis severity in previous studies [12, 13, 17]. In addition, we identified separately those who were dispensed ≥ 1 prescription of systemic corticosteroids, other immunosuppressants (methotrexate, azathioprine, leflunomide, or mycophenolate mofetil), non-steroidal anti-inflammatory drugs (used primarily for Löfgren’s syndrome among other indications), and inhaled corticosteroids within six months before diagnosis.

### Clinical variables from the Karolinska cohort

We had information on clinical variables at sarcoidosis diagnosis for a subset of patients diagnosed at Karolinska University Hospital by pulmonologists and consented to be included in our clinical cohort. Data included Löfgren’s syndrome at diagnosis (yes/no), Scadding stage (0–IV), elevated serum angiotensin converting enzyme (S-ACE; yes/no), HLA-DRB1 alleles (expression of at least one of *03, *07, *14, or *15) [18], percentage of predicted forced vital capacity (FVC) and diffusion capacity of the lungs for carbon monoxide (DLCO) based on Hedenström reference material [19, 20] that was used throughout the study period.

### Outcomes after sarcoidosis diagnosis

We and others showed that sarcoidosis is associated with a higher risk of several long-term outcomes and early death, especially in those requiring immunosuppressive treatment [12, 13, 17, 21, 22]. Therefore, we followed patients for adverse outcomes to examine whether the model-derived patient trajectories were associated with adverse outcomes. We searched for a new ICD-coded visit in the NPR after sarcoidosis diagnosis for an infectious disease, heart failure, diabetes, depression, and anxiety (see Table S1 for codes), or all-cause death in the National Cause of Death Register. Follow-up started at the second visit for sarcoidosis and ended at adverse outcome diagnosis, emigration, death, or December 31, 2023. Patients with a diagnosis of heart failure or diabetes at the second visit for sarcoidosis were excluded from the respective analysis.

### Statistical analysis

To model sarcoidosis trajectories, all patients were followed for five years from sarcoidosis diagnosis. Time since diagnosis was divided into 10 six-month intervals and for each interval we counted any ICD-coded visit for sarcoidosis in the NPR, allowing for one visit per day. We used zero-inflated Poisson models implemented in the command *traj* [23, 24] for Stata version 18 (StataCorp LLC, College Station, Texas, USA) to model patient trajectories based on accumulated visits during the 10 six-month intervals as polynomial functions of time since sarcoidosis diagnosis. Models with one to four trajectories were fit to visit data; the model with four trajectories did not converge. We selected the model with two trajectories as the main model based on model-fit statistics (Table S2) and visual inspection (Figure S1).

We examined associations between baseline sociodemographic and clinical variables outlined above and patient trajectories. We used modified Poisson regression models [25] estimating risk ratios (RR) and corresponding 95% confidence intervals (CI). We first ran univariable models and then a multivariable model aiming to identify which variables independently predicted patients’ trajectories considering the strength of associations. We performed similar analyses in a subset of patients included in the Karolinska cohort considering additional clinical variables. Missing data were marked and included as a separate category in all analyses except for the multivariable model for the Karolinska cohort including percentage predicted FVC and DLCO for which a complete-case analysis was performed.

To examine outcomes in patients by modelled trajectory, we estimated hazard ratios (HR) and corresponding 95% CIs for infectious disease, heart failure, diabetes, depression, anxiety, and death using Cox models adjusted for age at sarcoidosis diagnosis, sex, country of birth, region of residence, and education.

We conducted several secondary and sensitivity analyses. To ascertain whether model-derived trajectories reflected mostly visits for which sarcoidosis was the primary reason for the visit, we considered the proportion of patients with ≥ 1 visit where sarcoidosis was the primary discharge diagnosis and repeated trajectory analyses using only visits for which sarcoidosis was marked as the primary diagnosis. We repeated trajectory modeling with two trajectories restricting to visits for which sarcoidosis was coded as the primary diagnosis and examined predictors for chronic disease as in the primary analysis. We further examined predictors and outcomes for the model with three trajectories. Analysis for predictors associated with each trajectory was performed using a multinomial logistic regression model while outcome modeling was conducted using adjusted Cox regression models similar to the main analysis. In a final analysis, we modeled trajectories in patients that were treated around the time of diagnosis to account for treatment as the reason behind accumulation of healthcare visits.

Unless otherwise mentioned, data management, statistical analyses and visualizations were performed using SAS version 9.4 (SAS Institute Inc., Cary, NC, USA) and R version 4.5.3 (R Core Team, R Foundation for Statistical Computing, Vienna, Austria).

## Results

A total of 9665 individuals newly diagnosed with sarcoidosis between 2006 and 2018 were included in the trajectory analyses. The mean age at diagnosis was 50.4 years (standard deviation 14.2), and 43.2% were female. Additional demographic and clinical characteristics are shown in Table S3. About four in ten patients received immunosuppressant treatment around diagnosis and 26.0% were dispensed a prescription of non-steroidal anti-inflammatory drugs.

### Modelled patient trajectories

**Fig. 1:**
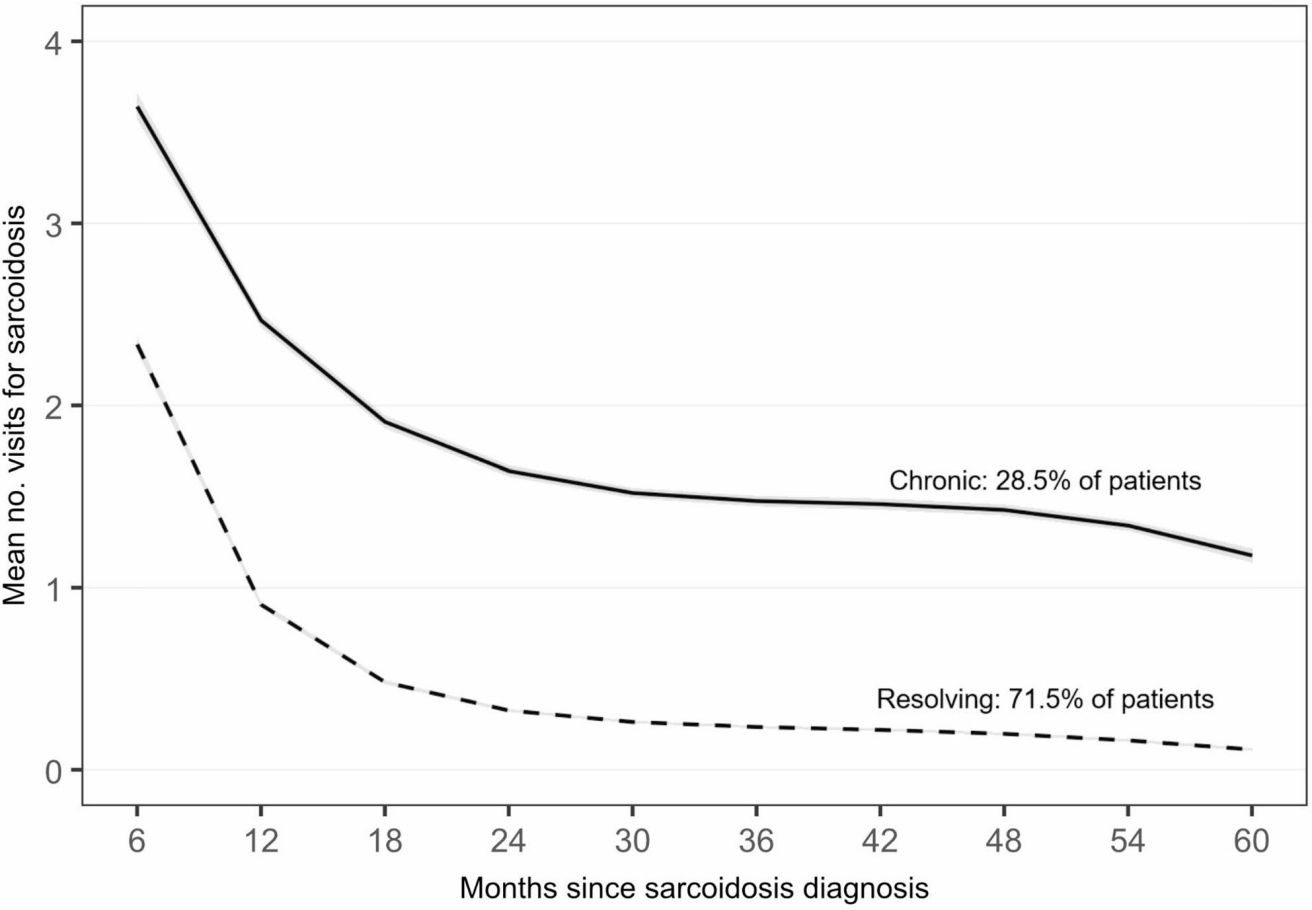
Plot shows modelled trajectories of patients with sarcoidosis based on the number of inpatient or outpatient visits in the National Patient Register listing an International Classification of Diseases code for sarcoidosis during six-month intervals after sarcoidosis diagnosis. Solid line: chronic sarcoidosis (28.5% of patients), dashed line: resolving sarcoidosis (71.5% of patients); line ribbon: 95% confidence interval

Patients were classified into two trajectories in the main model (Fig. 1). The larger group (*n* = 6909; 71.5% of patients) exhibited a rapid decline in sarcoidosis-related visits, reaching near zero within two years, consistent with resolving sarcoidosis. The smaller group (*n* = 2756; 28.5%) maintained persistently elevated visit frequencies, about 1.5 visits every six months after the second year, consistent with a chronic disease trajectory.

### Predictors of chronic sarcoidosis

Demographic and clinical characteristics stratified by trajectory are presented in Table 1. In multivariable models, older age, sex, Nordic country of birth, education, and civil status, had little to no impact on disease trajectory. Individuals diagnosed in the South and Southeast healthcare regions of Sweden and to a smaller extent those from the West were more likely to have a chronic disease course. Compared with others, the clinic of first diagnosis strongly predicted disease course; diagnoses in neurology (multivariable RR 1.57 [95% CI 1.30, 1.89]), ophthalmology (RR 1.54 [1.31, 1.80]), and cardiology (RR 1.23 [1.00, 1.52]) were associated with higher risks of chronic sarcoidosis. In contrast, diagnosis in rheumatology was associated with a 35% lower risk (RR 0.65 [0.53, 0.81]).

**Table 1:** Demographic and clinical characteristics of individuals with sarcoidosis stratified by clinical course of the disease within five years after diagnosis as modelled in the trajectory analysis. Risk ratios and corresponding 95% confidence intervals from univariate and multivariable modified Poisson regression models comparing chronic to resolving sarcoidosis

Factors evaluated at sarcoidosis diagnosis	Modelled sarcoidosis trajectory		Risk ratio (95% CI)	
	Chronic (*n* = 2756)	Resolving (*n* = 6909)	Univariable model	Multivariable model
Age^1^, years	51.7 (13.7)	49.8 (14.4)	1.07 (1.05, 1.09)	1.02 (1.00, 1.05)
Sex				
Female	1211 (43.9)	2962 (42.9)	1.03 (0.97, 1.10)	0.97 (0.91, 1.03)
Male	1545 (56.1)	3947 (57.1)	1.00 [Reference]	1.00 [Reference]
Region of residence				
Stockholm	520 (18.9)	1501 (21.7)	1.11 (0.97, 1.27)	1.19 (1.05, 1.35)
Uppsala-Örebro	544 (19.7)	1513 (21.9)	1.14 (1.00, 1.30)	1.08 (0.95, 1.23)
West	474 (17.2)	1235 (17.9)	1.20 (1.05, 1.37)	1.15 (1.01, 1.31)
South	595 (21.6)	1111 (16.1)	1.50 (1.32, 1.71)	1.40 (1.24, 1.59)
Southeast	380 (13.8)	745 (10.8)	1.46 (1.27, 1.67)	1.37 (1.20, 1.57)
North	243 (8.8)	804 (11.6)	1.00 [Reference]	1.00 [Reference]
Country of birth^2^				
Nordic	2451 (88.9)	6194 (89.7)	0.95 (0.86, 1.05)	0.96 (0.87, 1.06)
Non-Nordic	305 (11.1)	715 (10.3)	1.00 [Reference]	1.00 [Reference]
Education, years				
≤ 9	538 (19.5)	1196 (17.3)	1.10 (1.00, 1.20)	0.97 (0.89, 1.06)
10–12	1360 (49.3)	3523 (51.0)	0.99 (0.92, 1.06)	0.97 (0.90, 1.04)
≥ 13	841 (30.5)	2139 (31.0)	1.00 [Reference]	1.00 [Reference]
Missing	17 (0.6)	51 (0.7)	0.89 (0.58, 1.34)	0.86 (0.59, 1.26)
Civil status				
Married or registered partnership	1398 (50.7)	3300 (47.8)	1.00 [Reference]	1.00 [Reference]
Other	1358 (49.3)	3609 (52.2)	0.92 (0.86, 0.98)	0.94 (0.89, 1.00)
Clinic of first visit for sarcoidosis				
Respiratory Medicine	1036 (37.6)	3010 (43.6)	1.00 (0.87, 1.14)	1.11 (0.97, 1.27)
Rheumatology	92 (3.3)	396 (5.7)	0.73 (0.59, 0.92)	0.65 (0.53, 0.81)
Cardiology	65 (2.4)	86 (1.2)	1.68 (1.35, 2.09)	1.23 (1.00, 1.52)
Other Medicine	932 (33.8)	2157 (31.2)	1.18 (1.03, 1.34)	1.08 (0.94, 1.23)
Dermatology	118 (4.3)	425 (6.2)	0.85 (0.69, 1.03)	1.06 (0.87, 1.30)
Neurology	90 (3.3)	69 (1.0)	2.21 (1.84, 2.65)	1.57 (1.30, 1.89)
Ophthalmology	228 (8.3)	201 (2.9)	2.07 (1.78, 2.41)	1.54 (1.31, 1.80)
Other	195 (7.1)	565 (8.2)	1.00 [Reference]	1.00 [Reference]
Hospitalized at diagnosis	495 (18.0)	835 (12.1)	1.37 (1.27, 1.48)	1.19 (1.10, 1.30)
Family history of sarcoidosis^3^	86 (3.1)	228 (3.3)	0.96 (0.80, 1.15)	1.02 (0.86, 1.21)
> 2 healthcare visits within two years before diagnosis^4^	1990 (72.2)	4206 (60.9)	1.45 (1.35, 1.56)	1.22 (1.13, 1.32)
Morbidity at sarcoidosis diagnosis				
Myocardial infarction	68 (2.5)	126 (1.8)	1.24 (1.02, 1.50)	0.95 (0.78, 1.14)
Heart failure	100 (3.6)	137 (2.0)	1.50 (1.29, 1.74)	1.19 (1.01, 1.40)
Atrial fibrillation	105 (3.8)	215 (3.1)	1.16 (0.99, 1.36)	0.94 (0.80, 1.11)
Hypertension	905 (32.8)	1702 (24.6)	1.32 (1.24, 1.41)	1.15 (1.07, 1.25)
Diabetes mellitus	305 (11.1)	523 (7.6)	1.33 (1.21, 1.46)	1.10 (0.99, 1.21)
Chronic obstructive pulmonary disease	70 (2.5)	138 (2.0)	1.18 (0.98, 1.44)	0.95 (0.78, 1.15)
Asthma	174 (6.3)	356 (5.2)	1.16 (1.02, 1.32)	1.03 (0.90, 1.16)
Uveitis	400 (14.5)	473 (6.8)	1.71 (1.58, 1.85)	1.42 (1.29, 1.55)
Autoimmune disease	321 (11.6)	597 (8.6)	1.26 (1.14, 1.38)	1.08 (0.99, 1.19)
Immunodeficiency	16 (0.6)	24 (0.3)	1.41 (0.96, 2.06)	1.28 (0.90, 1.83)
Infectious disease within six months before diagnosis	228 (8.3)	489 (7.1)	1.13 (1.01, 1.26)	1.00 (0.90, 1.12)
Treated for sarcoidosis around diagnosis^5^	1691 (61.4)	2261 (32.7)	2.30 (2.15, 2.45)	2.18 (2.04, 2.33)
≥ 1 medication dispensation within six months before diagnosis				
Systemic corticosteroids	662 (24.0)	1099 (15.9)	1.42 (1.32, 1.52)	—
Other immunosuppressants	53 (1.9)	78 (1.1)	1.43 (1.16, 1.76)	—
Non-steroidal anti-inflammatory drugs	548 (19.9)	1969 (28.5)	0.70 (0.65, 0.76)	—
Inhaled corticosteroids	268 (9.7)	515 (7.5)	1.22 (1.10, 1.35)	—

Data are mean (standard deviation) or n (%) unless otherwise stated. Category percentages may not sum up to 100 owing to rounding

CI confidence interval

^1^ Effect estimated per 10-year increase in age

^2^ Nordic countries include Sweden, Norway, Denmark, Finland, and Iceland

^3^ History of having at least one first degree relative (biologic parents, full siblings, or offspring) with sarcoidosis at the time of sarcoidosis diagnosis

^4^ Includes hospitalizations, outpatient visits to specialists and day operations/surgeries

^5^ ≥1 dispensation of immunosuppressant treatment (systemic corticosteroids, methotrexate, or azathioprine) within three months before or after sarcoidosis diagnosis

Among comorbidities/manifestations at sarcoidosis diagnosis, uveitis (RR 1.42 [1.29, 1.55]), heart failure (RR 1.19 [1.01, 1.40]), and hypertension (RR 1.15 [1.07, 1.25]) predicted a chronic course (Table 1).

The strongest independent predictor was treatment around diagnosis, with patients dispensed an immunosuppressant (systemic corticosteroid, methotrexate, or azathioprine) within ± 3 months were more than twice as likely to develop chronic sarcoidosis (RR 2.18 [2.04, 2.33]; Table 1). Dispensation of a systemic corticosteroid within six months before diagnosis was associated with higher risk (RR 1.28 [1.19, 1.37]), whereas dispensation of a non-steroidal anti-inflammatory drug was associated with a lower risk (RR 0.76 [95% CI 0.70, 0.82]; Table S4).

In the Karolinska cohort, 166 patients (34.1%) were classified as chronic and 321 (65.9%) as of resolving course. Patients with Löfgren’s syndrome had a 53% lower risk of chronic disease (multivariable RR 0.47 [0.28, 0.77]), and carriers of the HLA-DRB1*03 allele showed a similarly reduced risk (RR 0.52 [0.34, 0.79]; Table 2). Patients classified as having chronic disease were slightly more likely to have advanced Scadding stage, elevated S-ACE levels, but the 95% CIs were wide due to limited numbers. Immunosuppressant treatment around diagnosis remained the strongest marker of chronic disease (RR 1.62 [1.29, 2.03]). In a subset of patients with available lung function tests (*n* = 252; 51.7%), a decrease in FVC by 5% points was associated with an 8% higher risk for chronicity (RR 1.08 [1.01, 1.15]; Table 2). No association was found with percentage predicted DLCO.

**Table 2:** Demographic and clinical characteristics of individuals with sarcoidosis registered in the Karolinska cohort stratified by disease course within five years after diagnosis (chronic vs. resolving) as modelled in the trajectory analysis. Risk ratios and corresponding 95% confidence intervals from multivariable modified Poisson regression models comparing chronic to resolving sarcoidosis

	Modelled sarcoidosis trajectory		Risk ratio (95% CI)	
	Chronic (*n* = 166)	Resolving (*n* = 321)	Multivariable model 1	Multivariable model 2
Age at diagnosis^1^, years	48.2 (11.4)	45.4 (12.2)	1.03 (0.94, 1.14)	1.00 (0.87, 1.15)
Sex				
Female	66 (39.8)	128 (39.9)	1.09 (0.86, 1.39)	1.27 (0.91, 1.78)
Male	100 (60.2)	193 (60.1)	1.00 [Reference]	1.00 [Reference]
Education, years				
≤ 9 or missing	24 (14.5)	25 (7.8)	1.39 (1.04, 1.87)	1.06 (0.68, 1.65)
10–12	67 (40.4)	136 (42.4)	1.13 (0.88, 1.45)	1.08 (0.77, 1.52)
≥ 13	75 (45.2)	160 (49.8)	1.00 [Reference]	1.00 [Reference]
Löfgren’s syndrome	18 (10.8)	129 (40.2)	0.47 (0.28, 0.77)	0.30 (0.15, 0.59)
Scadding stage				—^6^
0	8 (4.8)	12 (3.7)	1.00 [Reference]	
I	38 (22.9)	142 (44.2)	0.86 (0.51, 1.45)	
II	88 (53.0)	139 (43.3)	1.16 (0.72, 1.87)	
III	20 (12.0)	17 (5.3)	1.34 (0.77, 2.32)	
IV	10 (6.0)	6 (1.9)	1.70 (0.95, 3.05)	
Missing	2 (1.2)	5 (1.6)	1.03 (0.32, 3.31)	
S-ACE				
Elevated	49 (29.5)	68 (21.2)	1.10 (0.83, 1.45)	1.19 (0.85, 1.69)
Normal	70 (42.2)	178 (55.5)	1.00 [Reference]	1.00 [Reference]
Missing	47 (28.3)	75 (23.4)	1.08 (0.81, 1.43)	0.92 (0.56, 1.52)
HLA-DRB1 allele^2^				
*03	23 (13.9)	130 (40.5)	0.52 (0.34, 0.79)	0.58 (0.33, 0.99)
*07	25 (15.1)	22 (6.9)	1.14 (0.86, 1.51)	1.11 (0.74, 1.66)
*14	8 (4.8)	30 (9.3)	0.53 (0.31, 0.92)	0.48 (0.21, 1.11)
*15	68 (41.0)	98 (30.5)	0.98 (0.78, 1.24)	0.94 (0.69, 1.29)
FVC^3^, % predicted	83.8 (14.9)	89.0 (13.6)	—	1.08 (1.01, 1.15)
DLCO^4^, % predicted	84.7 (17.4)	88.8 (16.3)	—	0.96 (0.91, 1.02)
Treated sarcoidosis around diagnosis^5^	67 (40.4)	65 (20.2)	1.62 (1.29, 2.03)	1.45 (1.05, 2.01)

*CI* confidence interval, *S-ACE* serum angiotensin converting enzyme, *HLA* human leukocyte antigen, *FVC* forced vital capacity, *DLCO* diffusion capacity for carbon monoxide

Data are mean (standard deviation) or n (%) unless otherwise stated. Percentages may not sum up to 100 owing to rounding

^1^ Effect estimated per 10-year increase in age

^2^ At least one allele

^3^ Missing: Chronic, *n* = 43 (%); Resolving, *n* = 101 (%). Effect per 5% decrease

^4^ Missing: Chronic, *n* = 49 (%); Resolving, *n* = 97 (%). Effect per 5% decrease

^5^ ≥1 dispensation of immunosuppressant treatment (systemic corticosteroids, methotrexate, or azathioprine) within three months before or after sarcoidosis diagnosis

^6^ Not estimable with Scadding stage

### Select outcomes associated with chronic sarcoidosis

Chronic sarcoidosis was associated with increased hazards of all assessed outcomes (Table 3). Adjusted HRs comparing chronic versus resolving trajectories were 1.5–2.0-times higher; infection 1.62 (95% CI 1.51, 1.73), heart failure 1.99 (1.70, 2.32), diabetes mellitus 1.71 (1.52, 1.93), depression 1.58 (1.35, 1.87), anxiety disorder 1.47 (1.24, 1.74), and all-cause death 1.49 (1.30, 1.71).

**Table 3:** Adjusted hazard ratios and corresponding 95% confidence intervals of various outcomes observed in patients after sarcoidosis diagnosis comparing chronic to resolving sarcoidosis

Outcomes after sarcoidosis diagnosis	Model-derived sarcoidosis trajectory		Hazard ratio^1^ (95% CI)
	Chronic	Resolving	
Infectious disease	1327/2756 (48.1)	2315/6909 (33.5)	1.62 (1.51, 1.73)
Heart failure	284/2361 (10.8)	369/6737 (5.5)	1.99 (1.70, 2.32)
Diabetes mellitus	443/2436 (18.2)	688/6332 (10.9)	1.71 (1.52, 1.93)
Depression	231/2756 (8.4)	393/6909 (5.7)	1.58 (1.35, 1.87)
Anxiety disorder	208/2756 (7.5)	391/6909 (5.7)	1.47 (1.24, 1.74)
Death	317/2756 (11.5)	569/6909 (8.2)	1.49 (1.30, 1.71)

*CI* confidence interval

Data are N events/N at risk (%) unless otherwise stated

^1^ Hazard ratios were estimated from Cox proportional hazards models adjusted for age at sarcoidosis diagnosis, sex, region of residence, country of birth, and education

### Secondary and sensitivity analyses

Restricting to visits where sarcoidosis was the primary diagnosis yielded similar patterns (Figures S2 and S3) and yielded similar results in terms of predictors of chronic disease (Table S5). In a sensitivity analysis, a three-class model separated the resolving trajectory into two groups comprising patients with rapid (about 50%) and gradual/slow (approximately 42%) declines in sarcoidosis-related visits, while retaining a smaller chronic trajectory (8.4%) characterized by persistently elevated healthcare utilization (Figure S1). The principal distinction between patients with declining versus persistent healthcare utilization remained unchanged. In predictor and outcome analyses, we found similar results as in the main analysis, but with generally stronger associations for persistent chronic compared to slowly resolving and rapidly resolving sarcoidosis, respectively (Tables S6 and S7). Finaly, we found that a model with two classes classified patients treated around the time of sarcoidosis into slowly resolving and persistent chronic trajectories (approximately 65% and 35%, respectively; Figure S4).

## Discussion

This nationwide, data-driven trajectory cohort study demonstrated that sarcoidosis follows two distinct long-term courses, a resolving trajectory (71.5% of patients) characterized reduction of sarcoidosis-related healthcare use within two years, and a chronic trajectory (28.5%) with ongoing healthcare use for sarcoidosis over at least five years. Known clinical predictors such as sarcoidosis phenotype and treatment with an immunosuppressant around diagnosis emerged as strong predictors of clinical course. In addition, individuals in the chronic group had higher risks of adverse outcomes, including infection, cardiovascular disease, diabetes, mood disorders, and early death.

This is the first study to have used latent class modeling of group-based trajectories in a large and representative sarcoidosis cohort. Our analysis showed that although model fit statistics continued to improve with additional classes, the three-class trajectory model primarily subdivided the resolving trajectory according to the rate of decline in sarcoidosis-related visits rather than identifying an additional chronic disease phenotype. We therefore considered the two-class model to provide a more parsimonious representation of the clinically recognized distinction between resolving and chronic sarcoidosis. Our findings closely mirror those from clinical cohorts suggesting that spontaneous resolution of sarcoid inflammation irrespective of immunosuppressive treatment within two to five years after diagnosis is more common compared to chronic disease [4, 26–29].

Except for older age which was associated with a small increased risk of chronic sarcoidosis (8% risk-increase per decade of age), other demographic factors (i.e. sex and attained education) were not predictive of chronic sarcoidosis. Interestingly, we observed higher risk ratios of chronic sarcoidosis in individuals diagnosed in the southern, southeastern and western regions of Sweden. These finding may reflect subtle differences in sarcoidosis phenotypes and disease presentations [15], but also healthcare seeking patterns and/or diagnosis and treatment traditions among regions.

Phenotypic presentation emerged as a critical determinant of disease course. Patients diagnosed in rheumatology, a likely proxy for the acute presentation of Löfgren’s syndrome, had markedly better outcomes, consistent with its self-limiting nature. Approximately 30% of our patients present with Löfgren’s syndrome, which is known to have a favorable prognosis with most patients recovering within two years [4, 9, 11, 29]. Conversely, those diagnosed in ophthalmology, neurology, or cardiology, likely reflecting extrapulmonary manifestations in vital organs, had a significantly higher probability of chronic sarcoidosis. Indeed, ocular sarcoidosis was shown to precede diagnosis of systemic (extraocular) disease in many cases in an Australian cohort [30], and 60–90% of those in multiple cohorts will require treatment owing to progressive ocular inflammation [31–33]. More recently, a cluster analysis using phenotypic and treatment data from a large Spanish hospital showed that individuals with non-Löfgren’s pulmonary or extrapulmonary sarcoidosis manifestations (e.g., ocular, cardiac, renal) were less likely to archive remission five years after diagnosis, which was observed in 26% of patients [29].

The protective association of non-steroidal anti-inflammatory drug prescription dispensations and the strong association of immunosuppressant treatment with chronicity reinforce that clinical presentation and the need of treatment at diagnosis remain robust prognostic markers. In the absence of a severity index that covers all possible manifestations of sarcoidosis, we and others used treatment with an immunosuppressant around diagnosis (commonly an oral corticosteroid) to stratify patients according to disease severity and capture those with chronic and potentially progressing disease [12, 13, 29, 34, 35]. In the current study, we could confirm that dispensation of an immunosuppressant around the time of diagnosis was one of the strongest predictors of chronic disease. This finding is not surprising as immunosuppressants are recommended in cases who present with debilitating symptoms or life-threatening manifestations, or are at risk of disease progression, but is to be refrained from prescribing to patients presenting with Löfgren’s syndrome [1, 9].

In our study, treatment with immunosuppressants around diagnosis may have influenced at least in part patient classification into trajectories as this is often associated with healthcare utilization for medication management. However, as we showed in a secondary analysis, the persistence of distinct trajectories among patients receiving immunosuppressive treatment argues against treatment monitoring alone explaining the observed trajectory structure. Although healthcare encounters related to medication management likely contribute to visit frequency, substantial heterogeneity remained among treated patients, suggesting that the identified trajectories capture underlying differences in disease course beyond treatment status.

Findings from the Karolinska cohort validate these register-based patterns and previous findings. Carriage of HLA-DRB1*03 and presentation with Löfgren’s syndrome were strongly associated with a resolving course, consistent with prior genetic and clinical research [9, 11]. Lower lung function as approximated by the percentage predicted FVC, but not DLCO, was moderately associated with chronic disease course. Several explanations are possible. First, DLCO was available only in a subset of patients, resulting in limited statistical power. Second, DLCO is influenced by multiple physiological factors beyond severity of pulmonary sarcoidosis, including pulmonary vascular involvement, anemia, smoking status, and measurement variability. Third, our outcome reflected long-term healthcare utilization trajectories rather than exclusively pulmonary disease progression. Consequently, FVC may have better captured the burden of clinically significant pulmonary involvement associated with chronic disease in this cohort. Lastly, and in line with previous evidence [36], we found some indication that S-ACE and Scadding stage of pulmonary disease may predict patient trajectories. Overall, these observations suggest that genetic and phenotypic features may outperform some routine clinical measures in predicting disease persistence.

The pronounced burden of comorbid outcomes among patients with chronic sarcoidosis extends previous register studies that used treatment status as a marker of disease severity. Chronic patients had higher risks of infection, heart failure, diabetes, and eventually early death, underscoring the systemic impact of prolonged inflammation and/or therapy. We previously published similar findings using sarcoidosis treatment status around diagnosis as a predictor of severe and likely progressive sarcoidosis [12, 13, 17, 37]. Patients with a chronic course in our study were also more likely to be diagnosed with mental health disorders such as depression and anxiety disorder. Similar findings were reported in other cohorts [22, 38, 39]. The elevated risk of depression and anxiety highlights the psychological toll of long-term illness and supports integrating mental health evaluation into sarcoidosis management. We should note that outcome analyses were conducted to assess the prognostic validity of the data-driven trajectory classification rather than to estimate causal effects of sarcoidosis trajectory membership on subsequent clinical outcomes.

Our study has some noteworthy limitations. First, we did not have access to clinical information for the entire register-based cohort which limited power in trajectory predictor analyses. Second, without access to primary healthcare data and over the counter medication purchases, we may have underestimated some comorbidities and medication use (e.g., of non-steroidal anti-inflammatory drugs). Moreover, relying on data from secondary/tertiary care may have led to an overestimation of depression and anxiety disorder in the chronic group due to increased surveillance. Last, exclusion of individuals who died or emigrated within five years might have led to some underestimation of chronic cases [12]. Nevertheless, the large, population-based design and consistent results in our clinical cohort are considerable strengths and support the robustness and generalizability of our conclusions.

## Conclusions

In summary, in this large register-based cohort, sarcoidosis followed two trajectories: a resolving course possibly characterized by decline in healthcare use in approximately 70% of patients and a chronic course in about 30%. The phenotype of sarcoidosis, approximated by the clinic of diagnosis and the need for immunosuppressant treatment around diagnosis, strongly predicted chronic disease. Chronic sarcoidosis was associated with markedly higher risks of systemic and psychiatric comorbidities and premature mortality. These findings underscore the value of early clinical and treatment markers in predicting long-term outcomes and highlight the need for future studies integrating genetic and molecular data to improve personalized prognosis and management of sarcoidosis.

## Supplementary Information

Supplementary Material 1.

## Data Availability

The datasets used for the conduct of this study are covered by ethics and secrecy agreements and are not publicly available.

## Abbreviations

ATC: Anatomical Therapeutic Chemical
DLCO: Diffusion capacity for carbon monoxide
FVC: Forced vital capacity
HLA: Human leukocyte antigen
ICD: International Classification of Diseases
NPDR: National Prescribed Drug Register
NPR: National Patient Register
S-ACE: Serum angiotensin converting enzyme
