# Supplementary material for: Sarcoidosis data-driven patient trajectories and predictors of chronic disease
Source: Respir Res. 2026 Jul 24;27:292. doi: 10.1186/s12931-026-03830-z (PMC13397769; doi:10.1186/s12931-026-03830-z)
Supplement: Supplementary file 1 — Supplementary Material 1. [file 12931_2026_3830_MOESM1_ESM.docx]

|  | **SUPPLEMENTAL MATERIAL** |  |
| --- | --- | --- |

**Sarcoidosis data-driven patient trajectories and predictors of chronic disease**

Marios Rossides, Susanna Kullberg, Pernilla Darlington, Anders Eklund, Elizabeth V. Arkema

Contents

[SUPPLEMENTAL TABLES 2](#_Toc233538437)

[Table S1 2](#_Toc233538438)

[Table S2. 4](#_Toc233538439)

[Table S3 5](#_Toc233538440)

[Table S4 7](#_Toc233538441)

[Table S5 9](#_Toc233538442)

[Table S6 11](#_Toc233538443)

[Table S7 13](#_Toc233538444)

[SUPPLEMENTAL FIGURES 14](#_Toc233538445)

[Figure S1 14](#_Toc233538446)

[Figure S2 15](#_Toc233538447)

[Figure S3 16](#_Toc233538448)

[Figure S4 17](#_Toc233538449)

# SUPPLEMENTAL TABLES

Table S1. Variable definitions and codes.

| **Variable** | **Definition** | **Codes** |
| --- | --- | --- |
| Sarcoidosis  (including family history of sarcoidosis) | ≥2 inpatient or outpatient visits in the NPR | *ICD-8/9:* 135  *ICD-10:* D86 |
| Hematopoietic or lung malignancy | ≥1 mention in the National Cancer Register | *ICD-7:* 162–163; 200‒205 |
| Sarcoidosis immunosuppressant treatment around diagnosis | ≥1 dispensation in the NPDR ±3 months from the first sarcoidosis visit | *ATC:* H02AB01/02/04/06/07; L01BA01; L04AX01/03 |
| Myocardial infarction | ≥1 inpatient visit in the NPR | *ICD-8/9:* 410; 412  *ICD-10:* I21; I22; I25.2 |
| Heart failure | ≥1 inpatient or outpatient visit in the NPR | *ICD-8:* 425; 427,0; 428,9  *ICD-9:* 425; 428  *ICD-10:* I42; I50 |
| Atrial fibrillation | ≥1 inpatient or outpatient visit in the NPR | *ICD-8:* 427,92  *ICD-9:* 427D  *ICD-10:* I48 |
| Hypertension | ≥1 visit in the NPR or ≥2 dispensations in the NPDR | *ICD-8:* 401–404  *ICD-9:* 401–405  *ICD-10:* I10–I15  *ATC:* C07; C08; C09 |
| Diabetes mellitus | ≥1 visit in the NPR or ≥2 dispensations in the NPDR | *ICD-8/9:* 250  *ICD-10:* E10; E11  *ATC:* A10 |
| Chronic obstructive pulmonary disease | ≥1 inpatient or outpatient visit in the NPR | *ICD-8:* 491; 492  *ICD-9:* 491A; 491B; 491X; 492; 496  *ICD-10:* J41–J43; J44.1; J44.8; J44.9 |
| Asthma | ≥1 inpatient or outpatient visit in the NPR | *ICD-8/9:* 493  *ICD-10:* J45; J46 |
| Uveitis | ≥1 inpatient or outpatient visit in the NPR | *ICD-8/9:* 364  *ICD-10:* H20 |
| Autoimmune disease | ≥2 inpatient or outpatient visits in the NPR | *ICD-8:* 136,07; 242,00; 245,30; 255,10; 269,10; 281,0; 283,90; 287,10; 340; 357; 446,20; 446,30; 446,38; 563; 694; 696; 704,00; 712; 716; 733,00; 734,0; 734,1; 734,9  *ICD-9:* 136B; 242A; 245C; 255E; 281A; 283A; 287D; 340; 357A; 358A; 446E; 446F; 555; 556; 571G; 579A; 694E; 694F; 696; 704A; 710; 714; 720  *ICD-10:* D51.0; D59.1; D68.6; D69.3; E06.3; E05.0; E10; E27.1; G70.0; G35; G61.0; K50; K51; K74.3; K90.0; L10; L12; L40; L63; M05–M09; M31.3; M31.5; M31.6; M32.1; M32.8; M32.9; M33; M34; M35.0; M35.1; M35.2; M45 |
| Immunodeficiency (primary, non-acquired) | ≥1 inpatient or outpatient visit in the NPR | *ICD-9: 279J/L/M/X*  *ICD-10: D80–D84* |
| Infectious disease | ≥1 inpatient or outpatient visit in the NPR listing an ICD code for an infectious disease as the primary discharge diagnosis | *ICD-10:* A00–B99; D73.3; E06.0; E32.1; G00–G07; H00.0; H44.0; H60.0; H60.1; H60.2; H60.3; H66; H67; H70; I30.1; I40.0; J00–J22; J32; J34.0; J36; J38.3; J39.0; J39.1; J44.0; J85; J86; K04.4; K04.6; K04.7; K10.2; K11.3; K12.2; K14.0; K57.0; K57.2; K57.4; K57.8; K61; K63.0; K65.0; K65.9; L00–L08; L30.3; M00; M01; M46.2; M46.3; M46.4; M60.0; M64.5; M65.0; M71.0; M71.1; M72.6; M86; N10–N12; N13.6; N15.1; N15.9; N30.0; N30.8; N34.0; N39.0; N41.2; N43.1; N45; N48.2; N61; N70–N74; N75.1; O23; O26.4; O41.1; O75.3; O85; O86; O88.3; O91; O98 |
| Depression | ≥1 inpatient or outpatient visit in the NPR | *ICD-10:* F32; F33 |
| Anxiety | ≥1 inpatient or outpatient visit in the NPR | *ICD-10:* F40; F41 |
| Systemic corticosteroids | ≥1 dispensation in the NPDR | *ATC:* H02AB01/02/04/06/07 |
| Other immunosuppressants | ≥1 dispensation in the NPDR | *ATC:* L01BA01; L04AX03; L04AX01; L04AA13 |
| Non-steroidal anti-inflammatory drugs | ≥1 dispensation in the NPDR | *ATC:* M01A |
| Inhaled corticosteroids | ≥1 dispensation in the NPDR | *ATC:* R03BA |
| NPR = National Patient Register; NPDR = National Prescribed Drug Register; ICD = International Classification of Diseases; ATC = Anatomical Therapeutic Chemical. | | |

Table S2. Model fit statistics and size of each class (modelled trajectory) of the zero-inflated Poisson models that were tested.

| **No. classes** | **Model parametrization of each trajectory class (group)** | **Minimum class size, %** | **Model-fit statistics^1^** | | | | |
| --- | --- | --- | --- | --- | --- | --- | --- |
|  |  |  | **Bayesian Information Criterion (BIC)** | **Akaike Information Criterion (AIC)** | **Log-likelihood** | **Entropy** | **Average posterior probabilities** |
| 1 | Cubic | 100.0 | -121583 | -121568 | -121564 | — | — |
| 2 | Cubic; cubic | 28.5 | -106352 | -106320 | -106311 | 0.88 | 0.972; 0.945 |
| 3 | Quadratic; quadratic; quadratic | 8.4 | -103832 | -103793 | -103782 | 0.84 | 0.916; 0.937; 0.917 |
| 4 | Not estimable | | | | | | |
| ^1^ Model-fit statistics depend on the number of estimated parameters, that is, the number of classes and type of parametrization of each class (cubic > quadratic > linear). | | | | | | | |

Table S3. Demographic and clinical characteristics of individuals with sarcoidosis.

| **Factors evaluated at sarcoidosis diagnosis** | **All patients**  (n=9665) |
| --- | --- |
| Age, years | 50.4 (14.2) |
| Sex |  |
| Female | 4173 (43.2) |
| Male | 5492 (56.8) |
| Region of residence |  |
| Stockholm | 2021 (20.9) |
| Uppsala-Örebro | 2057 (21.3) |
| West | 1709 (17.7) |
| South | 1706 (17.7) |
| Southeast | 1125 (11.6) |
| North | 1047 (10.8) |
| Country of birth^1^ |  |
| Nordic | 8645 (89.4) |
| Non-Nordic | 1020 (10.6) |
| Education, years |  |
| ≤9 | 1734 (17.9) |
| 10–12 | 4883 (50.5) |
| ≥13 | 2980 (30.8) |
| Missing | 68 (0.7) |
| Civil status |  |
| Married or registered partnership | 4698 (48.6) |
| Other | 4967 (51.4) |
| Clinic of first visit for sarcoidosis |  |
| Respiratory Medicine | 4046 (41.9) |
| Rheumatology | 488 (5.0) |
| Cardiology | 151 (1.6) |
| Other Medicine | 3089 (32.0) |
| Dermatology | 543 (5.6) |
| Neurology | 159 (1.6) |
| Ophthalmology | 429 (4.4) |
| Other | 760 (7.9) |
| Hospitalized at diagnosis | 1330 (13.8) |
| Family history of sarcoidosis^2^ | 314 (3.2) |
| >2 healthcare visits within two years before diagnosis^3^ | 6196 (64.1) |
| Morbidity at sarcoidosis diagnosis |  |
| Myocardial infarction | 194 (2.0) |
| Heart failure | 237 (2.5) |
| Atrial fibrillation | 320 (3.3) |
| Hypertension | 2607 (27.0) |
| Diabetes mellitus | 828 (8.6) |
| Chronic obstructive pulmonary disease | 208 (2.2) |
| Asthma | 530 (5.5) |
| Uveitis | 873 (9.0) |
| Autoimmune disease | 918 (9.5) |
| Immunodeficiency | 40 (0.4) |
| Infectious disease within six months before diagnosis | 717 (7.4) |
| Treated for sarcoidosis around diagnosis^4^ | 3952 (40.9) |
| ≥1 medication dispensation within six months before diagnosis |  |
| Systemic corticosteroids | 1761 (18.2) |
| Other immunosuppressants | 131 (1.4) |
| Non-steroidal anti-inflammatory drugs | 2517 (26.0) |
| Inhaled corticosteroids | 783 (8.1) |
| Data are mean (standard deviation) or n (%). Percentages may not sum up to 100 owing to rounding.  ^1^ Nordic countries include Sweden, Norway, Denmark, Finland, and Iceland.  ^2^ History of having at least one first degree relative (biologic parent, full sibling, or biologic offspring) with sarcoidosis at the time of sarcoidosis diagnosis.  ^3^ Includes hospitalizations and/or outpatient visits to secondary/tertiary care.  ^4^ ≥1 dispensation of immunosuppressant treatment (systemic corticosteroids, methotrexate, or azathioprine) within three months before or after sarcoidosis diagnosis. | |

Table S4. Risk ratios and corresponding 95% confidence intervals from a multivariable modified Poisson regression model comparing chronic to resolving sarcoidosis.

| **Factors evaluated at sarcoidosis diagnosis** | **Risk ratio (95% CI)** |
| --- | --- |
| Age^1^, years | 1.00 (0.97, 1.02) |
| Sex |  |
| Female | 0.97 (0.91, 1.04) |
| Male | 1.00 [Reference] |
| Region of residence |  |
| Stockholm | 1.16 (1.02, 1.32) |
| Uppsala-Örebro | 1.15 (1.01, 1.31) |
| West | 1.17 (1.03, 1.34) |
| South | 1.44 (1.27, 1.64) |
| Southeast | 1.49 (1.30, 1.71) |
| North | 1.00 [Reference] |
| Country of birth |  |
| Nordic | 0.95 (0.86, 1.06) |
| Non-Nordic | 1.00 [Reference] |
| Education, years |  |
| ≤9 | 1.00 (0.92, 1.10) |
| 10–12 | 1.00 (0.93, 1.07) |
| ≥13 | 1.00 [Reference] |
| Missing | 0.94 (0.88, 1.00) |
| Civil status |  |
| Married or registered partnership | 1.00 [Reference] |
| Other | 0.95 (0.89, 1.02) |
| Clinic of first visit for sarcoidosis |  |
| Respiratory Medicine | 1.16 (1.01, 1.33) |
| Rheumatology | 0.90 (0.72, 1.11) |
| Cardiology | 1.47 (1.18, 1.84) |
| Other Medicine | 1.23 (1.07, 1.40) |
| Dermatology | 1.00 (0.81, 1.22) |
| Neurology | 2.01 (1.67, 2.43) |
| Ophthalmology | 1.75 (1.48, 2.06) |
| Other | 1.00 [Reference] |
| Hospitalized at diagnosis | 1.39 (1.27, 1.51) |
| Family history of sarcoidosis^2^ | 0.96 (0.80, 1.15) |
| >2 healthcare visits within two years before diagnosis^3^ | 1.23 (1.14, 1.33) |
| Morbidity at sarcoidosis diagnosis |  |
| Myocardial infarction | 0.90 (0.74, 1.10) |
| Heart failure | 1.19 (1.01, 1.41) |
| Atrial fibrillation | 0.89 (0.75, 1.05) |
| Hypertension | 1.19 (1.10, 1.29) |
| Diabetes mellitus | 1.10 (0.99, 1.22) |
| Chronic obstructive pulmonary disease | 1.00 (0.82, 1.22) |
| Asthma | 1.01 (0.89, 1.15) |
| Uveitis | 1.41 (1.28, 1.55) |
| Autoimmune disease | 1.12 (1.02, 1.24) |
| Immunodeficiency | 1.29 (0.90, 1.87) |
| Infectious disease within six months before diagnosis | 1.02 (0.91, 1.14) |
| ≥1 medication dispensation within six months before diagnosis |  |
| Systemic corticosteroids | 1.28 (1.19, 1.37) |
| Other immunosuppressants | 0.99 (0.80, 1.23) |
| Non-steroidal anti-inflammatory drugs | 0.76 (0.70, 0.82) |
| Inhaled corticosteroids | 1.25 (1.13, 1.39) |
| CI = confidence interval.  ^1^ Effect estimated per 10-year increase in age.  ^2^ History of having at least one first degree relative (biologic parents, full siblings, offspring) with sarcoidosis at the time of sarcoidosis diagnosis.  ^3^ Includes hospitalizations and/or outpatient visits to secondary/tertiary care. | |

Table S5. Risk ratios and corresponding 95% confidence intervals from a multivariable modified Poisson regression model comparing chronic to resolving sarcoidosis restricted to visits for which sarcoidosis was the primary diagnosis.

| **Factors evaluated at sarcoidosis diagnosis** | **Risk ratio (95% CI)** |
| --- | --- |
| Age^1^, years | 1.01 (0.98, 1.03) |
| Sex |  |
| Female | 0.95 (0.90, 1.01) |
| Male | 1.00 [Reference] |
| Region of residence |  |
| Stockholm | 1.13 (1.01, 1.28) |
| Uppsala-Örebro | 0.98 (0.87, 1.10) |
| West | 1.04 (0.92, 1.18) |
| South | 1.37 (1.22, 1.54) |
| Southeast | 1.15 (1.01, 1.30) |
| North | 1.00 [Reference] |
| Country of birth |  |
| Nordic | 0.95 (0.86, 1.04) |
| Non-Nordic | 1.00 [Reference] |
| Education, years |  |
| ≤9 | 0.91 (0.83, 0.99) |
| 10–12 | 0.97 (0.90, 1.03) |
| ≥13 | 1.00 [Reference] |
| Missing | 0.79 (0.53, 1.16) |
| Civil status |  |
| Married or registered partnership | 1.00 [Reference] |
| Other | 0.91 (0.86, 0.97) |
| Clinic of first visit for sarcoidosis |  |
| Respiratory Medicine | 1.14 (1.00, 1.29) |
| Rheumatology | 0.64 (0.52, 0.79) |
| Cardiology | 1.21 (0.98, 1.49) |
| Other Medicine | 1.07 (0.94, 1.21) |
| Dermatology | 1.00 (0.82, 1.22) |
| Neurology | 1.49 (1.24, 1.79) |
| Ophthalmology | 1.13 (0.95, 1.35) |
| Other | 1.00 [Reference] |
| Hospitalized at diagnosis | 1.07 (0.98, 1.16) |
| Family history of sarcoidosis^2^ | 1.06 (0.90, 1.24) |
| >2 healthcare visits within two years before diagnosis^3^ | 1.10 (1.02, 1.18) |
| Morbidity at sarcoidosis diagnosis |  |
| Myocardial infarction | 0.84 (0.67, 1.06) |
| Heart failure | 0.98 (0.80, 1.20) |
| Atrial fibrillation | 0.81 (0.67, 0.98) |
| Hypertension | 1.06 (0.98, 1.14) |
| Diabetes mellitus | 1.02 (0.91, 1.14) |
| Chronic obstructive pulmonary disease | 0.91 (0.74, 1.12) |
| Asthma | 1.00 (0.88, 1.13) |
| Uveitis | 1.34 (1.22, 1.48) |
| Autoimmune disease | 0.90 (0.81, 1.00) |
| Immunodeficiency | 0.80 (0.49, 1.30) |
| Infectious disease within six months before diagnosis | 1.02 (0.92, 1.14) |
| Treated for sarcoidosis around diagnosis^4^ | 2.20 (2.07, 2.35) |
| CI = confidence interval.  ^1^ Effect estimated per 10-year increase in age.  ^2^ History of having at least one first degree relative (biologic parents, full siblings, offspring) with sarcoidosis at the time of sarcoidosis diagnosis.  ^3^ Includes hospitalizations and/or outpatient visits to secondary/tertiary care.  ^4^ ≥1 dispensation of immunosuppressant treatment (systemic corticosteroids, methotrexate, or azathioprine) within three months before or after sarcoidosis diagnosis. | |

Table S6. Odds ratios and corresponding 95% confidence intervals from a multivariable multinomial Logistic regression model comparing chronic and gradually/slowly resolving to rapidly resolving sarcoidosis.

| **Factors evaluated at sarcoidosis diagnosis** | **Odds ratio (95% CI)** | |
| --- | --- | --- |
|  | **Chronic** | **Slowly resolving** |
| Age^1^, years | 1.05 (0.98, 1.12) | 1.11 (1.07, 1.15) |
| Sex |  |  |
| Female | 1.12 (0.95, 1.33) | 0.85 (0.78, 0.94) |
| Male | 1.00 [Reference] | 1.00 [Reference] |
| Region of residence |  |  |
| Stockholm | 1.56 (1.12, 2.17) | 0.76 (0.65, 0.90) |
| Uppsala-Örebro | 1.07 (0.77, 1.50) | 0.88 (0.75, 1.03) |
| West | 1.14 (0.81, 1.61) | 0.90 (0.76, 1.06) |
| South | 2.25 (1.61, 3.13) | 1.18 (0.99, 1.40) |
| Southeast | 1.73 (1.20, 2.49) | 1.23 (1.03, 1.48) |
| North | 1.00 [Reference] | 1.00 [Reference] |
| Country of birth |  |  |
| Nordic | 1.00 (0.77, 1.29) | 1.07 (0.92, 1.24) |
| Non-Nordic | 1.00 [Reference] | 1.00 [Reference] |
| Education, years |  |  |
| ≤9 | 0.96 (0.76, 1.21) | 0.89 (0.78, 1.01) |
| 10–12 | 0.94 (0.78, 1.13) | 0.98 (0.89, 1.09) |
| ≥13 | 1.00 [Reference] | 1.00 [Reference] |
| Missing | 1.26 (0.54, 2.92) | 0.78 (0.45, 1.35) |
| Civil status |  |  |
| Married or registered partnership | 1.00 [Reference] | 1.00 [Reference] |
| Other | 0.81 (0.69, 0.96) | 0.93 (0.85, 1.02) |
| Clinic of first visit for sarcoidosis |  |  |
| Respiratory Medicine | 1.09 (0.78, 1.51) | 1.30 (1.09, 1.56) |
| Rheumatology | 0.65 (0.41, 1.02) | 0.46 (0.35, 0.61) |
| Cardiology | 2.00 (1.13, 3.52) | 1.39 (0.93, 2.08) |
| Other Medicine | 0.97 (0.70, 1.33) | 1.10 (0.93, 1.32) |
| Dermatology | 0.81 (0.49, 1.34) | 0.75 (0.58, 0.96) |
| Neurology | 3.14 (1.86, 5.30) | 1.37 (0.91, 2.07) |
| Ophthalmology | 3.11 (2.06, 4.70) | 0.86 (0.64, 1.16) |
| Other | 1.00 [Reference] | 1.00 [Reference] |
| Hospitalized at diagnosis | 1.63 (1.30, 2.04) | 1.02 (0.89, 1.18) |
| Family history of sarcoidosis^2^ | 1.39 (0.91, 2.12) | 1.02 (0.80, 1.31) |
| >2 healthcare visits within two years before diagnosis^3^ | 1.53 (1.26, 1.86) | 1.25 (1.13, 1.38) |
| Morbidity at sarcoidosis diagnosis |  |  |
| Myocardial infarction | 0.92 (0.54, 1.57) | 0.88 (0.64, 1.22) |
| Heart failure | 1.54 (0.99, 2.38) | 0.84 (0.62, 1.14) |
| Atrial fibrillation | 1.03 (0.67, 1.57) | 0.89 (0.68, 1.15) |
| Hypertension | 1.32 (1.08, 1.63) | 1.13 (1.00, 1.27) |
| Diabetes mellitus | 1.25 (0.95, 1.66) | 1.16 (0.98, 1.38) |
| Chronic obstructive pulmonary disease | 0.58 (0.32, 1.04) | 0.92 (0.68, 1.25) |
| Asthma | 1.41 (1.03, 1.93) | 1.03 (0.85, 1.25) |
| Uveitis | 2.97 (2.33, 3.80) | 1.54 (1.29, 1.83) |
| Autoimmune disease | 1.04 (0.80, 1.34) | 1.02 (0.88, 1.20) |
| Immunodeficiency | 1.20 (0.37, 3.91) | 1.24 (0.63, 2.43) |
| Infectious disease within six months before diagnosis | 1.24 (0.94, 1.63) | 0.96 (0.81, 1.14) |
| Treated for sarcoidosis around diagnosis^4^ | 4.87 (4.10, 5.80) | 2.41 (2.19, 2.64) |
| CI = confidence interval.  ^1^ Effect estimated per 10-year increase in age.  ^2^ History of having at least one first degree relative (biologic parents, full siblings, offspring) with sarcoidosis at the time of sarcoidosis diagnosis.  ^3^ Includes hospitalizations and/or outpatient visits to secondary/tertiary care.  ^4^ ≥1 dispensation of immunosuppressant treatment (systemic corticosteroids, methotrexate, or azathioprine) within three months before or after sarcoidosis diagnosis. | | |

Table S7. Adjusted hazard ratios and corresponding 95% confidence intervals of various outcomes observed in patients after sarcoidosis diagnosis comparing chronic and gradually/slowly resolving to rapidly resolving sarcoidosis.

| **Outcomes after sarcoidosis diagnosis** | **Hazard ratio^1^**  **(95% CI)** | | |
| --- | --- | --- | --- |
|  | **Chronic** | **Slowly resolving** | **Rapidly resolving** |
| Infectious disease | 2.12 (1.91, 2.36) | 1.29 (1.20, 1.38) | 1.00 [Reference] |
| Heart failure | 2.50 (1.97, 3.17) | 1.34 (1.14, 1.59) | 1.00 [Reference] |
| Diabetes mellitus | 2.14 (1.78, 2.57) | 1.18 (1.04, 1.34) | 1.00 [Reference] |
| Depression | 1.81 (1.40, 2.33) | 1.26 (1.06, 1.49) | 1.00 [Reference] |
| Anxiety disorder | 1.74 (1.35, 2.25) | 1.17 (0.98, 1.39) | 1.00 [Reference] |
| Death | 2.05 (1.66, 2.53) | 1.07 (0.93, 1.23) | 1.00 [Reference] |
| CI = confidence interval.  ^1^ Hazard ratios were estimated from Cox proportional hazards models adjusted for age at sarcoidosis diagnosis, sex, region of residence, country of birth, and education. | | | |

# SUPPLEMENTAL FIGURES


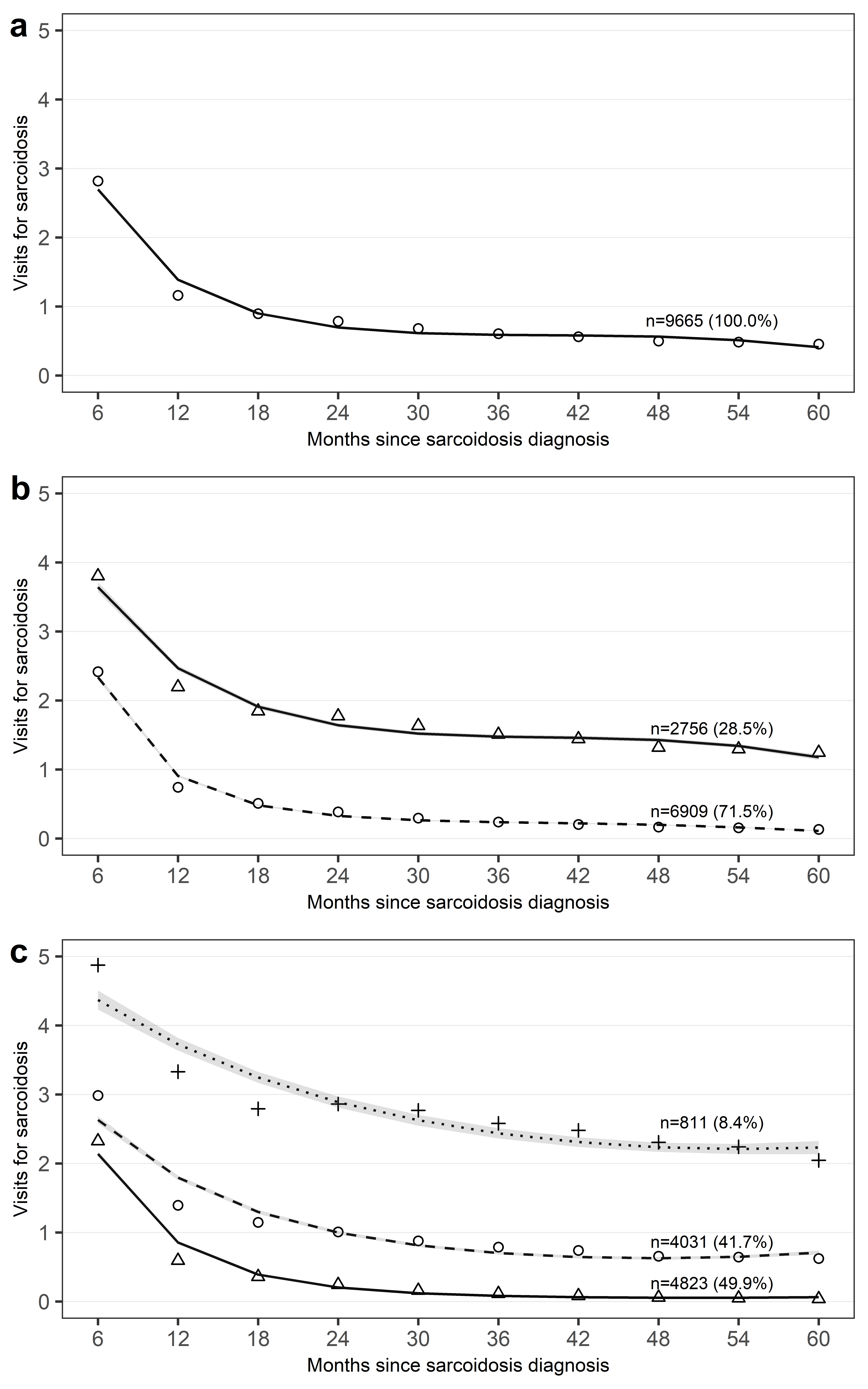


Figure S1. Plots show results of finite mixture modelling with (a) one, (b) two, and (c) three classes (group trajectories) using visits in the National Patient Register listing an ICD code for sarcoidosis. “n (%)” refers to the number of patients allocated in each modal class and percentage of total number of patients.


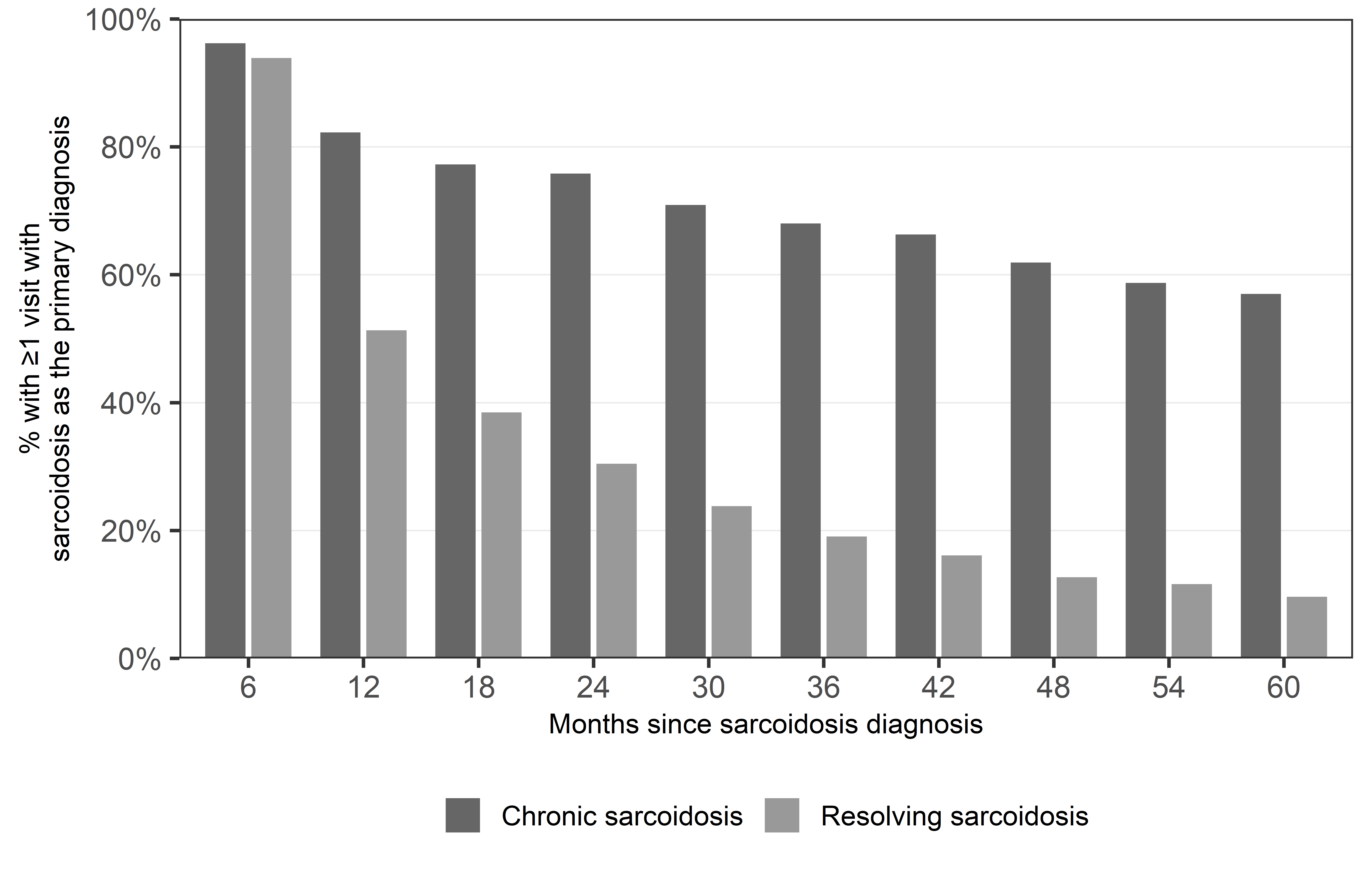


Figure S2. Plot shows percentage of patients with at least one visit listing an International Classification of Diseases code for sarcoidosis as the primary discharge diagnosis stratified by modelled sarcoidosis trajectory (chronic vs. resolving).


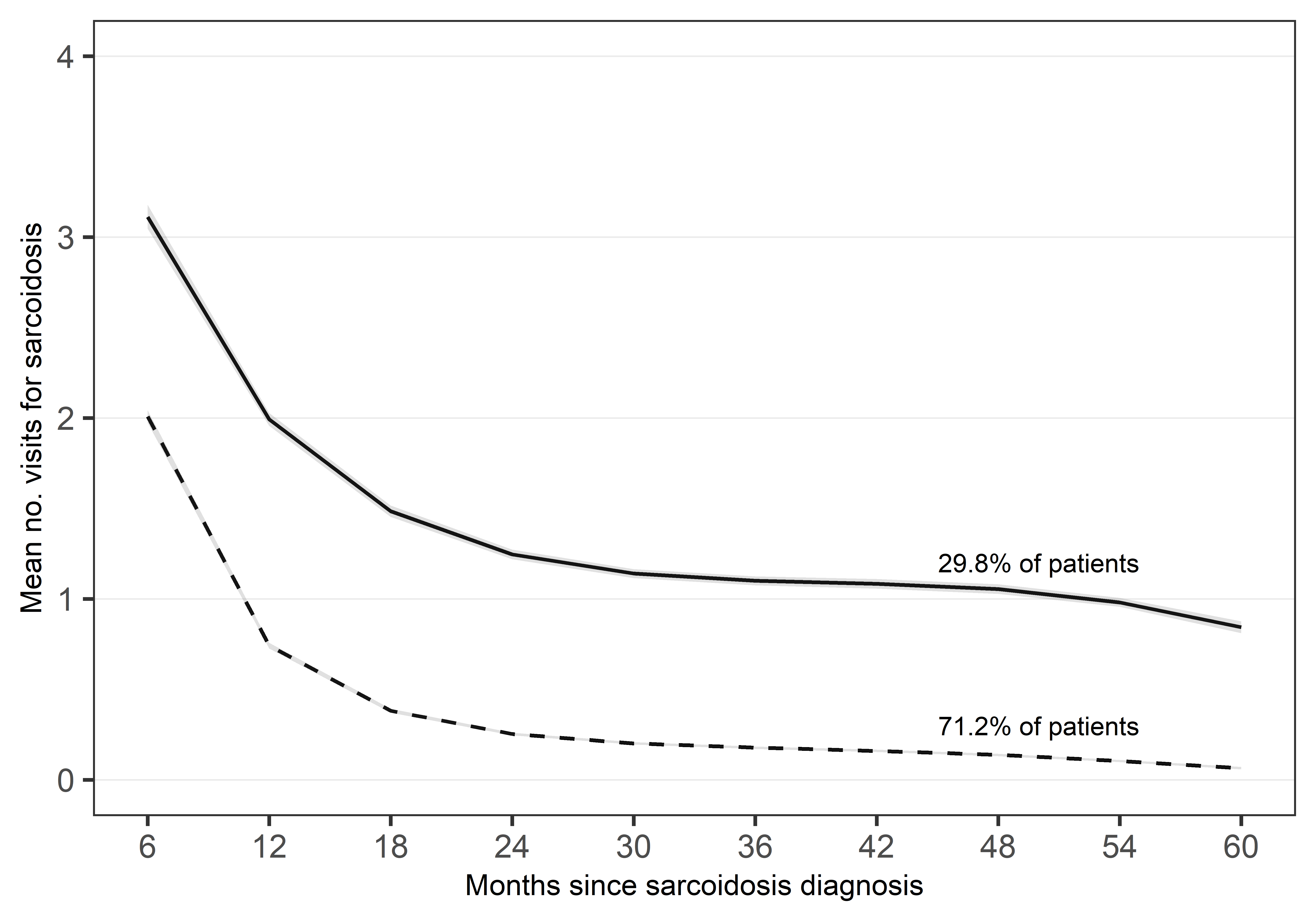


Figure S3. Plot shows modelled trajectories of patients with sarcoidosis based on the number of inpatient or outpatient visits in the National Patient Register listing an International Classification of Diseases code for sarcoidosis as the primary diagnosis during six-month intervals after sarcoidosis diagnosis.


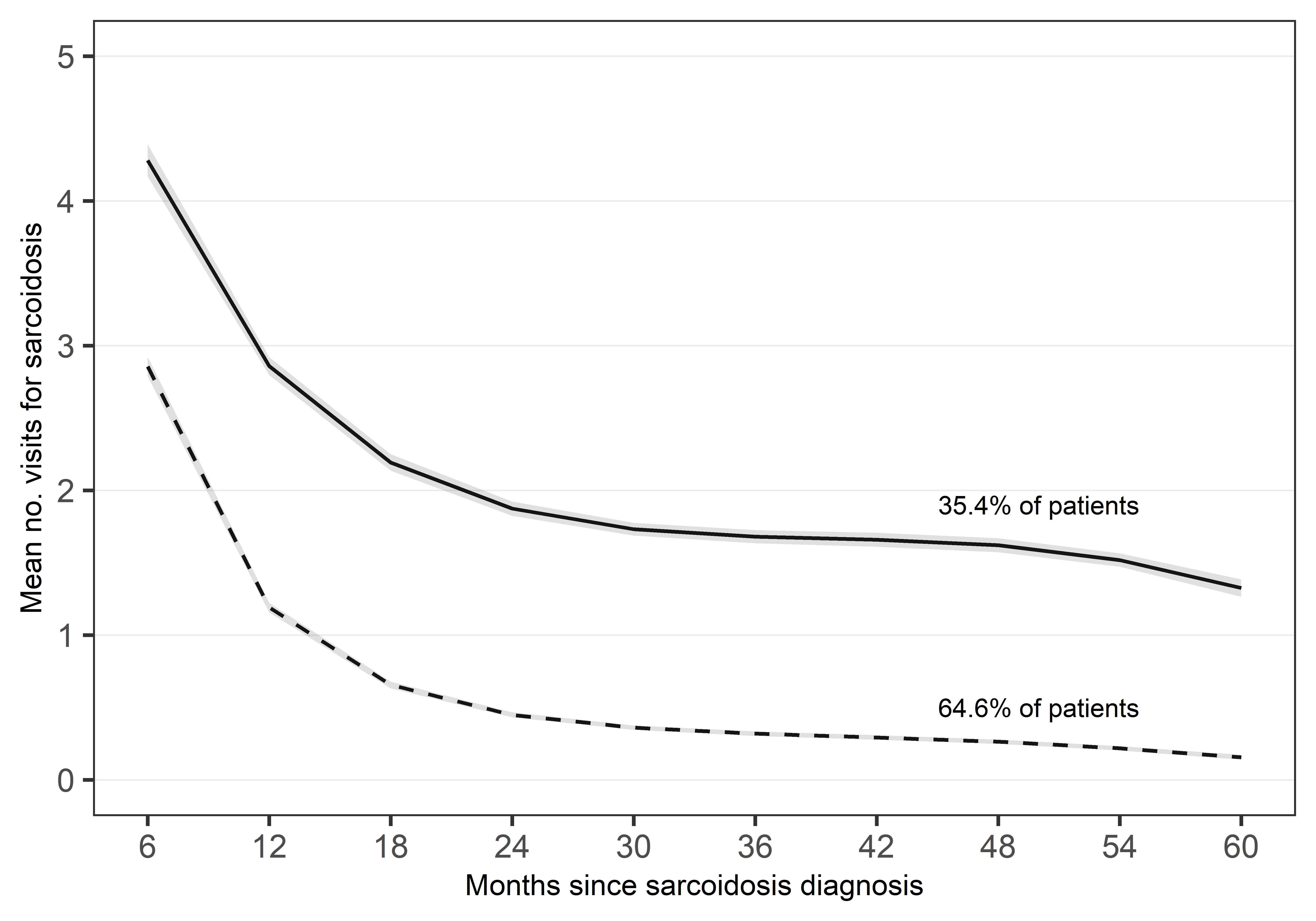


Figure S4. Plot shows modelled trajectories of patients with sarcoidosis treated around the time of diagnosis (see Methods section) based on the number of inpatient or outpatient visits in the National Patient Register listing an International Classification of Diseases code for sarcoidosis during six-month intervals after sarcoidosis diagnosis.
